# Physicochemical properties and bioavailability of naturally formulated fat‐soluble vitamins extracted from agricultural products for complementary use for natural vitamin supplements

**DOI:** 10.1002/fsn3.1804

**Published:** 2020-09-08

**Authors:** Hyun Jeong Lee, Changho Shin, Yoon Seok Chun, Jongkyu Kim, Hansang Jung, Jaijun Choung, Soon Mi Shim

**Affiliations:** ^1^ Department of Food Science and Biotechnology Sejong University Seoul Republic of Korea; ^2^ Department of Sports Science Sungkyunkwan University Gyeonggi‐do Republic of Korea; ^3^ Aribio Co., Ltd. Gyeonggi‐do Republic of Korea; ^4^ Department of Physical Education Kangnam University Gyeonggi‐do Republic of Korea

**Keywords:** antioxidant, bioavailability, shelf life, vitamins

## Abstract

The purpose of the current study was to evaluate the physicochemical properties, digestive stability, storage stability, and intestinal absorption of formulated natural vitamins (FNV) by mixing fat‐soluble vitamins extracted from agricultural products with their synthetic vitamin (SYNV) counterparts using a 6 to 4 ratio (w:w, dry weight). The FNV A, D, E, and K were evenly dispersed without crystal growth in the dispersion specifications for the functional tablet foods. The FNV A, D, E, and K had 89, 73, 65, and 36% of the digestive recovery, respectively, which was comparable to that of the SYNV. FNV D, E, and K were retained over 77%, but rapidly decreased to 15% after 6 months during accelerated storage at 25 30 and 35℃. The comparable radical scavenging capacity was found between the FNV and the SYNV. Results from the current study suggest that fat‐soluble vitamins extracted from agricultural products could be reasonable complementary use for natural vitamin supplements.

## Graphical abstract

Fat‐soluble vitamins are necessary for regulating various cell metabolism including anti‐oxidation, cell signaling, and modulation of the immune system. Consumers prefer vitamin supplements containing natural additives to synthetic additives with regard to efficacy and safety. However, the natural substance has drawbacks on actual biological use due to low stability during processing and storage and cost‐wise. This study evaluated the type of naturally formulated vitamin dispersion, bio‐accessibility, intestinal absorption, and radical scavenging capacity.

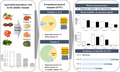

## 1 INTRODUCTION

Vitamins are essential to human body, and 13 vitamins have been identified and classified as fat‐soluble vitamins (A, E, D, and K) and water‐soluble vitamins (B‐group vitamins and vitamin C) according to their solubility (Turner & Mathiasson, 2000). In particular, fat‐soluble vitamins are necessary for regulating various cell metabolisms, which include anti‐oxidation, cell signaling, and the modulation of the immune system (Chadare et al., 2019; Turner, King, & Mathiasson, 2001). Vitamin A and its precursor, which is β‐carotene, are predominantly found in plants, and they are mainly necessary for protecting the retina as well as scavenging free radicals (Akhtar & Bryan, 2008; Widjaja‐Adhi, Lobo, Golczak, & Von Lintig, 2015). Vitamin D consists of two different compounds, which include vitamin D_2_ and D_3_, and it controls the cell proliferation and regulates calcium and bone mineralization (Miller, 2017; Qiu et al., 2020). Vitamin E is the group name of closely related tocopherols, which are the biological antioxidants and the regulators of the gene expression (Niki, 2014). Vitamin K, which is essential for the bone metabolism and the blood coagulation, is general term of both K_1_ (phylloquinone) and K_2_ (menaquinone) (Schurgers et al., 2006). Vitamin K_1_ is 90% of the dietary vitamin K, and it is mostly synthesized in green plant leaves (Takada et al., 2015). Vitamins A, D, E, and K are predominantly distributed in carrots, mushrooms, tomatoes, and spinach. (Akhtar & Bryan, 2008; Bügel, Spagner, Poulsen, Jakobsen, & Astrup, 2016; Simon, Phillips, Horst, & Munro, 2011).

**Figure 1:**
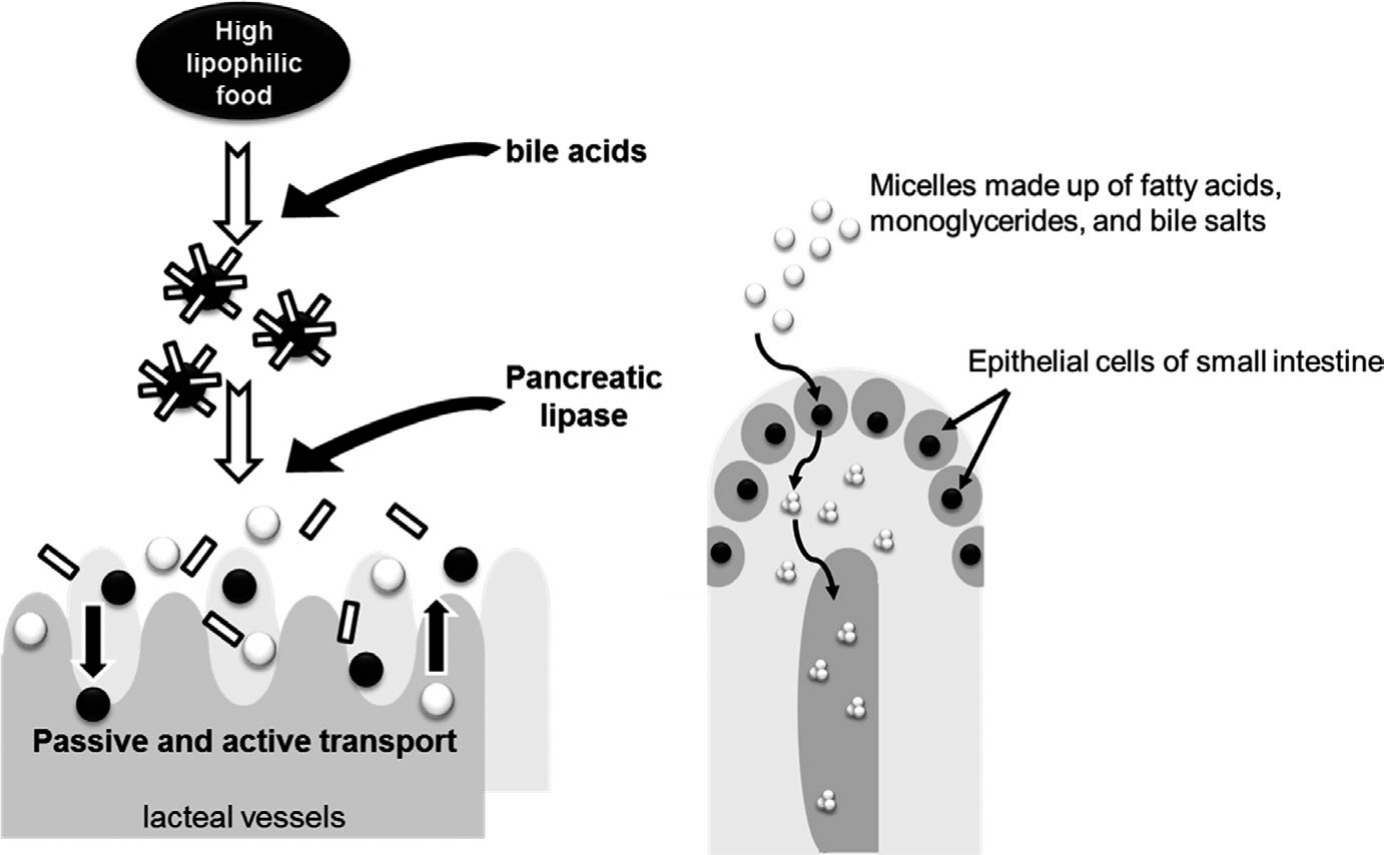
Digestion and intestinal metabolism of high lipophilic food microconstituents, such as fat‐soluble vitamins

The metabolism of high lipophilic food microconstituents, such as fat‐soluble vitamins, can start in the stomach where the foods are exposed to acidic conditions and gastric enzymes (Campbell, 2017). In contrast to water‐soluble vitamins, fat‐soluble vitamins are not readily absorbed and are required to interact with bile acid and pancreatic lipase in digestive fluids, which is shown in Figure 1 (Goncalves et al., 2015). In detail, vitamin A was frequently esterified and hydrolyzed to retinol and fatty acids. The free retinol was then integrated with other lipids into the formed micelles, and it was absorbed by the brush border membranes of the intestine cells (Kiokias, Proestos, & Varzakas, 2016). After digestion, the fat‐soluble vitamins are typically absorbed at particular locations in the small intestine, and they are incorporated with the digestion of ingested dietary fats and oils (Goncalves et al., 2015; Wang et al., 2019). Once they are micellized, the physiological regulators in the intestinal membrane are responsible for their absorption (Schubert et al., 2018; Takada et al., 2015). For instance, vitamin D is absorbed by the passive and the active transport mechanisms (Reboul et al., 2011). The absorbed fat‐soluble vitamins join into chylomicrons are activated in the liver and then they are released into the lymphatic circulation system (Porter & Charman, 1997). Fat‐soluble vitamins generally have poor water solubility during digestion, and encapsulation within in the lipid‐based delivery systems is suggested in order to improve their bio‐accessibility. In contrast to vitamins A, D, and E, the intestinal absorption regarding the physiological regulator for vitamin K has not been well‐documented.

Since vitamins are not synthesized or formed from inappropriate amounts from the human body, they have to be ingested as outsourced food and nutritional supplements in order to prevent various diseases related to their deficiencies (Challem, 1999). A previous study found that consumers prefer vitamin supplements that contain natural additives to synthetic additives in regard to efficacy and safety (Devcich, Pedersen, & Petrie, 2007; Topliss et al., 2002). However, natural substances have drawbacks with the actual biological uses due to low stability during processing and storage (Pokorný, 2007). For instance, the biological activity of natural antioxidants is typically poorer than the biological activity of synthetic antioxidants, because natural antioxidants are not pure ingredients, and there is a dearth of information on their safety (Pokorný, 2007). In order to overcome these limitations, complementary use between the natural and the synthetic compounds as an approach for new vitamin supplements has been previously proposed. To be suitable as dietary fat‐soluble vitamin supplements that originated from natural substances intended for human intake, these types of formulations must be enabled in order to be solubilized and absorbed from the digestive system and enterocyte beyond their stability. Furthermore, the stability of fat‐soluble vitamins loaded in re‐assembled casein micelles was mostly affected by the conditions of the storage (Loewen, Chan, & Li‐Chan, 2018). It is plausible that their biological activities, such as radical scavenging activity, could be affected by the digestive and the storage stability once it is added into food or supplements. Thus, the purpose of the current study was to evaluate the physicochemical properties, which include the stability in the pH, dispersion, the storage stability, and the antioxidant activity as well as to estimate the digestive stability and the intestinal absorption of the natural fat‐soluble vitamins formulated with vitamins extracted from the agricultural products.

## 2 MATERIALS AND METHODS

### 2.1 Chemicals and reagents

Pyrogallol (≥98% of purity, HPLC grade), potassium hydroxide (reagent grade, 90%), benzene (≥99.9% of purity, HPLC grade), and chloroform (≥99% of purity, HPLC grade) were purchased from Sigma‐Aldrich (St. Louis, MO, USA). Acetonitrile and methyl alcohol of HPLC grade were purchased from J. T. Baker Chemical Co (Phillipsburg, NJ, USA). Ethyl alcohol (HPLC grade), petroleum ether (Extra Pure‐grade), dichloromethane (99.8% of purity, HPLC grade), and n‐hexane (HPLC grade) were purchased from Daejung chemical Co (Ltd Gyeonggi‐do, Republic of Korea). Standards with HPLC grade of β‐carotene, retinyl acetate, ergocalciferol (D_2_), cholecalciferol (D_3_), DL‐alpha‐tocopherol acetate, and phylloquinone (K_1_) were purchased from Sigma‐Aldrich. All chemicals used were of analytical‐reagent grade. The synthetic vitamins (SYNV) A, D, and E were purchased by DSM Nutritional Products LTD. In detail, SYNV A is composed of vitamin A acetate (11.5%), maltodextrin (47%), DL‐alpha‐tocopherol (1.5%), acacia (20%), and corn starch (20%). SYNV D is made of vitamin D3 crystalline (0.25%), acacia gum (38%), sucrose (38%), medium‐chain triglycerides (7.5%), corn starch (15.55%), and silicon dioxide (0.5%). The SYNV E is made up of DL‐alpha‐tocopherol (50%), modified food starch (24.5%), maltodextrin (24.5%), and silicon dioxide (1%). SYNV K consisted with vitamin K (5%), sucrose (5%), and acacia (90%) was obtained from Shaanxi Pioneer Biotech Co. Ltd.

### 2.2 Sample preparations

The standard stock solution of the vitamins was prepared and stored in a dark at room temperature (+1~ −13 ℃) until further analysis. The lyophilized powders of the carrots, tomatoes, mushrooms, and spinach were supplied by Aribio. The extraction yield of the freeze‐dried powder of the carrots, tomatoes, mushrooms, and spinach was 9.96%, 6.03%, 10.74%, and 10.35%, respectively. The extraction of the fat‐soluble vitamins from each powder was carried out by the method described in the previous studies (Ostermeyer & Schmidt, 2006; Turner & Mathiasson, 2000). In detail, the freeze‐dried powder of the carrots and the tomatoes was weighed in a round bottom flask. 260 ml of ethanol, 10 ml of pyrogallol ethanol solution, and 30 ml of 90% potassium hydroxide solution were added into it. The mixture went through saponification for 2 hr in a water bath at 65℃, and then it was quickly cooled, which was followed adding 300 ml of water. After that, it was transferred to a 2,000 ml separator funnel. 300 ml of petroleum ether was added into the separator funnel, and it was then gently shaken for 30 min. The petroleum ether layer was collected into a separate flask, and the compound was then re‐extracted with 300 ml of petroleum ether, which was followed by collection once again as above. Both of the petroleum ether layers were concentrated under vacuum pressure from 40℃ to 50℃. The final solution was diluted one time with distilled water, and it was then filtered through 0.45‐μm membrane filters.

The aliquot amount of mushrooms (30 g) was taken in a round bottom flask and mixed with 260 ml of ethanol, 10 ml of pyrogallol ethanol solution, and 30 ml of 90% potassium hydroxide solution. The same method was applied as described above. According to a previous study (Jäpelt & Jakobsen, 2016), approximately 60 g of spinach weighed into a separator funnel was mixed with 240 ml of methanol and 400 ml of dichloromethane (V: V, 1:1.6), and it was then shaken 5 times. After 1 hr, the supernatant from the liquid layer was transferred to a new distillation flask, and a mixture of methanol and dichloromethane (V: V, 1:1.6) was added for shaking. After 1 hr, both of the liquid extracts were pooled together and then passed through Whatman filter paper No. 42 (MF‐Millipore). The supernatant liquid layer collected after the filtering was concentrated under vacuum pressure from 40℃ to 50℃. The final solution was diluted with distilled water (V: V, 1:1) and filtered through 0.45‐μm membrane filters.

### 2.3 Formulation of tablet for natural vitamins

Formulated natural vitamins (FNV), which include vitamins A, D, E, and K, were prepared by Aribio Company. In detail, natural vitamins A, D, and E and extracted powder from agricultural products were mixed with synthetic vitamins (SYNV, 100% of chemicals) purchased from DSM Nutritional Products (LTD, Kaiseraugst, Switzerland) at the ratio of 60% to 40% in a dry weight base where 1% of magnesium stearate and 1% of silicon dioxide were mixed in order to make a tablet. In the case of vitamin K, it was formulated with a 25% mixture of synthetic vitamins, the vitamins extracted powder from agricultural products (4:6, w: w, dry weight), 1% of magnesium stearate, 1% of silicon dioxide, and 73% of crystalline cellulose. The aliquot amount (2.4 g) of each formulated natural vitamin (FNV) was used for further experiments.

### 2.4 Identification and quantification of a vitamin

High‐performance liquid chromatography (HPLC) with a photodiode‐array detector (PDA) (Waters) that was equipped with an isocratic pump, a degasser, and automatic injection system (Model Ultimate 3000, Thermo Science) was used. The column used in this study was a YMC‐Pack ODS‐A C18 (150 mm x 4.6 mm, 5 μm). For the analysis of the β‐carotene, the analytical column was maintained at a temperature of 22 ± 1 ℃. The mobile phase consisted of a mixture of methyl tertiary‐butyl ether (MTBE) with methanol (60:40, v/v). The flow rate was set to 2 ml/min with a UV absorbance of 450 nm. To analyze the retinal acetate, ergocalciferol (D_2_), cholecalciferol (D_3_), DL‐alpha‐tocopherol acetate, and phylloquinone (K_1_), and the temperature of column was maintained at 30 ± 1℃. Acetonitrile (ACN) was used as a mobile phase. The flow rate was maintained at 1 ml/min with a UV absorbance of 280 nm (Fanali, D'Orazio, Fanali, & Gentili, 2017). The chromatographic peaks for each vitamin were identified by comparing the retention times of the samples with those of the standard compounds. Quantification was conducted using a standard calibration equation obtained from each external standard.

### 2.5 Measurement of the water solubility

The experiments on the water solubility of the functional tablet food made of the agricultural products were conducted by the method developed in the Korean Food Standards Codex (Korea Ministry of Food and Drug Safety, 2018a) and in 1907 in the Edition of Pharmacopoeia Helvetica (Al‐Gousous & Langguth, 2015). In order to measure the water solubility of the FNV A, D, E, and K, an aliquot amount of each FNV (2.4 g) was dispersed in 24 ml of distilled water and then was shaken for 30 min at 100 rpm in a water bath that was maintained at 37℃.

### 2.6 Measurements of digestive stability and intestinal uptake

The digestive stability and the intestinal cellular uptake of the FNV A, D, E, and K were estimated by using the in vitro bio‐mimic system coupled with a caco‐2 cell as described by Jeong et al. (2018)with slight modifications. The small intestinal phase of digestion was initiated adjusting to a pH 2.0 ± 0.1 by 0.1M NaHCO3 and 4 ml of pancreatin (2 mg/ml PB), lipase (1 mg/ml PB), and bile acid (12 mg/ml PB) were added. After being treated in a Caco‐2 cell, the obtained cells were cultured at 37℃ for 30 min and sonicated for 30 min. The sonicated cells were centrifuged at 13,000 rpm for 5 min at 4℃. The supernatant was filtered through a PTFE syringe filter (Advantec, pore size 0.45 μm) and stored at −20℃ until the analysis.

### 2.7 Prediction of shelf life

Formulated natural vitamins (FNV) A, D, E, and K were stored in a temperature/humidity‐controlled chamber (HANBAEK) at base temperatures (30 ± 1 ℃) and two temperatures (25 ± 1 ℃ and 35 ± 1 ℃) for comparison. The relative humidity was set at a constant value of 90%. The FNV A, D, E, and K were kept at three temperatures of 25 ± 1℃, 30 ± 1℃, and 35 ± 1℃ and were stored for 6 months. After 1.2, 2.4, 3.6, 4.9, and 6 months, the stored samples were taken out in order to measure the contents of the FNV A, D, E, and K. For a detailed prediction of the shelf life, the calculation was borrowed from other studies (Korea Ministry of Food and Drug Safety, 2018b; Jafari, Ganje, Dehnad, Ghanbari, & Hajitabar, 2017). The prediction of the shelf life was performed based on the rate equation of a degradation reaction by the Arrhenius model. The Arrhenius model for all process was simulated by the zero‐order model, the first‐order model, the Arrhenius equation, and the shelf‐life prediction (Li et al., 2019). The safety faction (0.8) was applied to determine the final shelf life of the FNV A, D, E, and K estimated for a temperature of 30℃.

### 2.8 Measurement of oxygen radical absorbance capacity (ORAC)

The antioxidant capability was measured using the oxygen radical absorbance capacity (ORAC) assay described by (Son, Choi, Kim, Park, & Shim, 2016). The ORAC values were converted as micromolar Trolox equivalents (TE) per 1 mg of sample (TE µmol/g). The curves of the relative fluorescence intensity of vitamins A, D, E, and K from the FNV and the SYNV were expressed as the net area under curve (Net AUC = AUC of Fluorescence sample − AUC of Fluorescence blank).

### 2.9 Statistical analysis

All the values were performed in triplicate. The analysis of variance (ANOVA) with the Tukey's post hoc test at a significant level of *p* < .05 was conducted using GraphPad Prism 3.0 software (GraphPad Software).

## 3 RESULTS AND DISCUSSION

### 3.1 Determination of fat‐soluble vitamins from selected agricultural products

**Figure 2:**
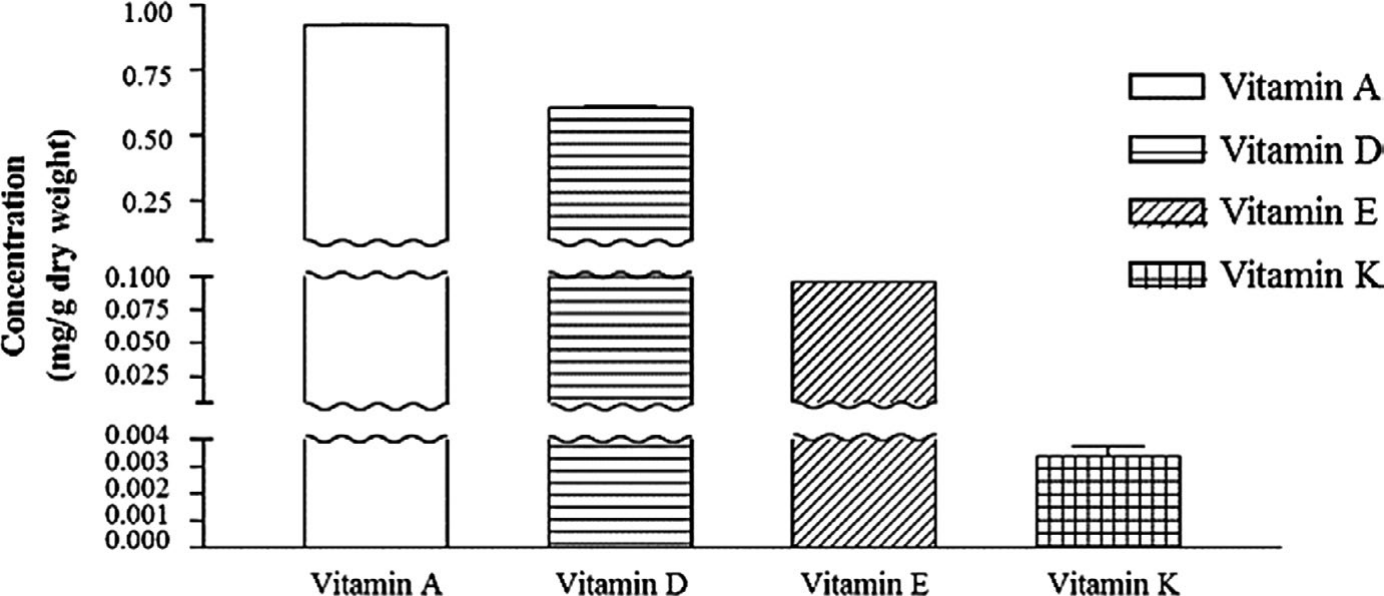
Identification and quantification of fat‐soluble vitamins (A, D, E, and K) by HPLC‐UV. The amount of vitamin extraction from the agricultural products, such as carrots, mushrooms, tomatoes, and spinach. The data include the means ± the *SEM* of the triplicated samples

The retention time (Rt) of each fat‐soluble vitamin standard simultaneously identified was as follows: β‐carotene = 1.216 min, alpha‐tocopherol acetate = 17.610 min, ergosterol = 22.550 min, and phylloquinone = 25.068 min (data not shown). Each standard curve from the β‐carotene, ergosterol, alpha‐tocopherol acetate, and phylloquinone was used for the quantification of each vitamins A, D, E, and K, respectively. Fat‐soluble vitamins including vitamin A, D, E, and K extracted from selected agricultural products such as carrots, mushrooms, tomatoes, and spinach, respectively, were also identified and quantified by comparison of its retention time and standard curve, respectively. The amount of vitamin A from carrots, vitamin D from tomatoes, vitamin E from mushrooms, and vitamin K from spinach was determined to be 0.921 (3,066.242 IU/g), 0.606 (2018.541 IU/g), 0.0955, and 0.0035 mg/g of dry weight, respectively (Figure 2). The content (0.921 mg/g of dry weight) of vitamin A obtained from the carrots, which was calculated by the β‐carotene, was higher than the levels obtained by the previous studies (0.789 mg/g of dry weight and 0.038 ~ 0.079 mg/g fresh weight, respectively) (Rawson, Tiwari, Tuohy, O’Donnell, & Brunton, 2011; Zaccari, Cabrera, Ramos, & Saadoun, 2015). A previous study found that the quantity of vitamin D_2_ in mushrooms calculated from ergosterol was 0.0268 ~ 0.458 mg/g of dry weight (Gąsecka, Magdziak, Siwulski, & Mleczek, 2018), which was comparable to the result of the current study (0.606 mg/g of dry weight). The amount of vitamin E based on the standard calibration of alpha‐tocopherol acetate was 0.0955 mg/g dry weight in tomatoes, which was higher than that of a previous finding (0.06 mg/g dry weight) from Raiola et al., (2015). Vitamin K_1_ calculated by phylloquinone amounted to 0.0035 mg/g dry weight. It was about 806 times higher than vitamin K_1_ in fresh spinach (4,340 ug/100 g of dry weight of spinach), which was reported by Jäpelt and Jakobsen (2016).

At the same time, the current study estimated the contribution of the consumption of each representative agricultural product for each of the fat‐soluble vitamins to the dietary reference intake (DRI) for Koreans or the adequate intake (AI) (Ministry of Health & Welfare, 2015). The DRI for vitamin A is 690 µg retinol activity equivalents (RAE)/day, and the AI for vitamins D, E and K is 11, 11.8, and 71 µg/day, respectively. In the current study, the determined amount of vitamin A from the agricultural products was equal to 1.32% of the DRI, and vitamins D, E, and K reached to 60%, 0.008%, and 0.05% of the AI, respectively (data not shown). The results from the current study imply that carrots, mushrooms, tomatoes, and spinach could provide good natural sources of vitamins A, D, E and K, respectively. Based on these results, the current study further examined the physicochemical properties and the bioavailability of the fat‐soluble vitamin extracts from the agricultural products to see the possibility of complementary use as natural vitamin supplements.

### 3.2 Dispersion of fat‐soluble vitamins for functional tablet foods

**Figure 3:**
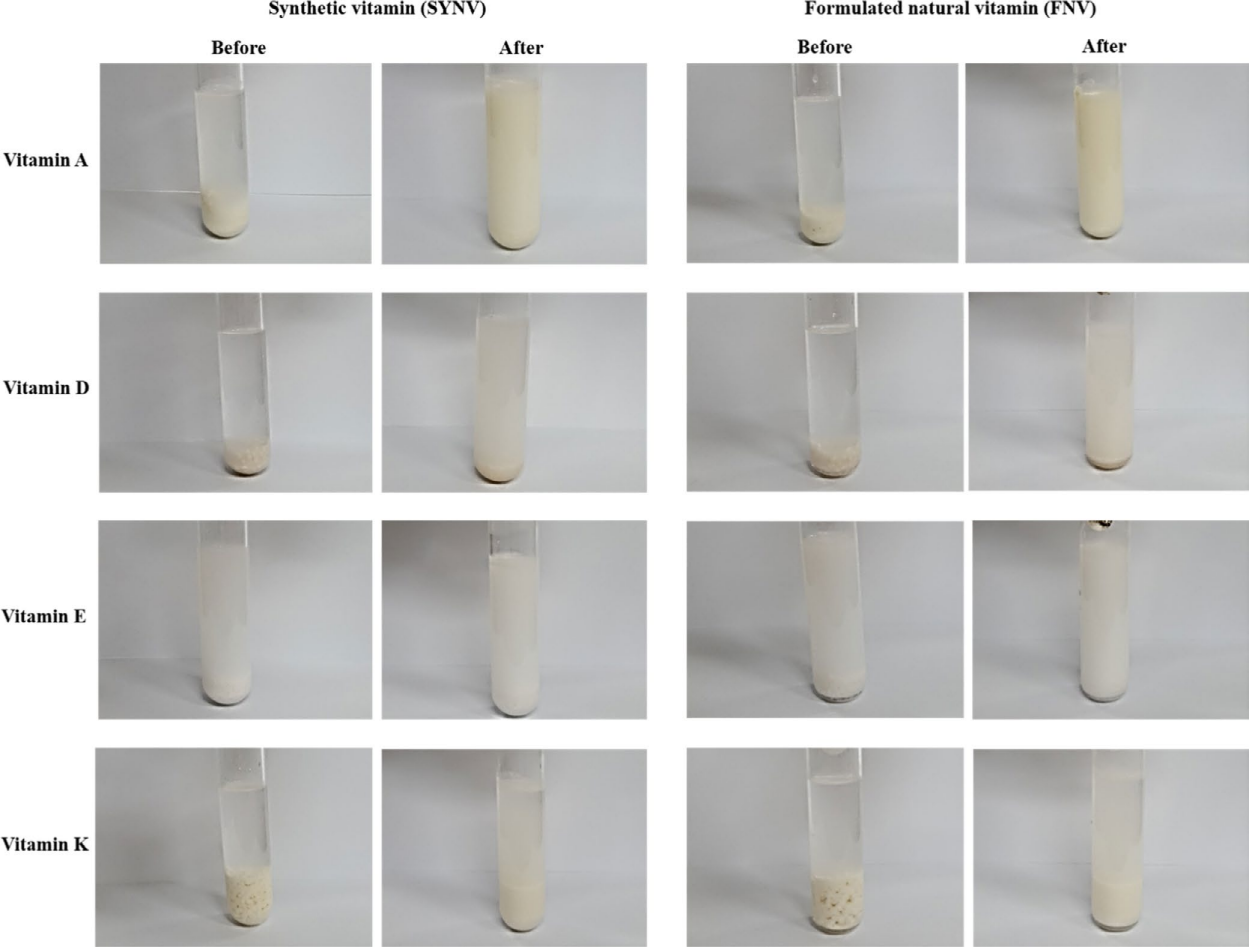
Dispersion test‐left: before and after dispersing SYNV; right: before and after dispersing FNV A, D, E, and K in the water at 37℃ for 30 min

Dispersion is a physical process related to mechanically decomposing tablets or capsules into small particles, and a tablet orally administered should be dissolved in the gastrointestinal tract fluid before absorption (Krsteska, Kostovski, Brzilova, Sejfulah, & Ugarkovic, 2010). To ensure that the active ingredient in the FNV A, D, E, and K is released efficiently from a formulated tablet and is then decomposed, the dispersion test was conducted as described in the materials and the methods. In the case of the FNV (A and E) and the SYNV (A and E), it was evenly dispersed without any crystal growth, and there was no significant difference between the natural and synthetic one (*p* > .05) (Figure 3). In contrast, vitamins D and K for both the FNV and the SYNV were initially dispersed in water without forming crystals, but they were gradually precipitated within 30 min after solubilizing in water at 37℃ (Figure 3). The results from the current study indicated that all single‐tablet formulation fully dispersed in water at 37℃ within 30 min and met the dispersion time requirement of the regulation. Thus, the FNV A, D, E, and K formulated with vitamins extracted from the agricultural products followed by mixing the synthetic vitamins could be within the dispersion specifications. The rate of dissolution influenced the absorption rate and the efficacy of the tablet products, which significantly influenced its bioavailability (Silva, Webster, Bou‐Chacra, & Löbenberg, 2018). Hence, the current study subsequently evaluated the bio‐accessibility and the intestinal absorption in the gastrointestinal tract fluid by using an in vitro biomimetic system.

### 3.3 Bio‐accessibility and intestinal cellular uptake of FNV

**Figure 4:**
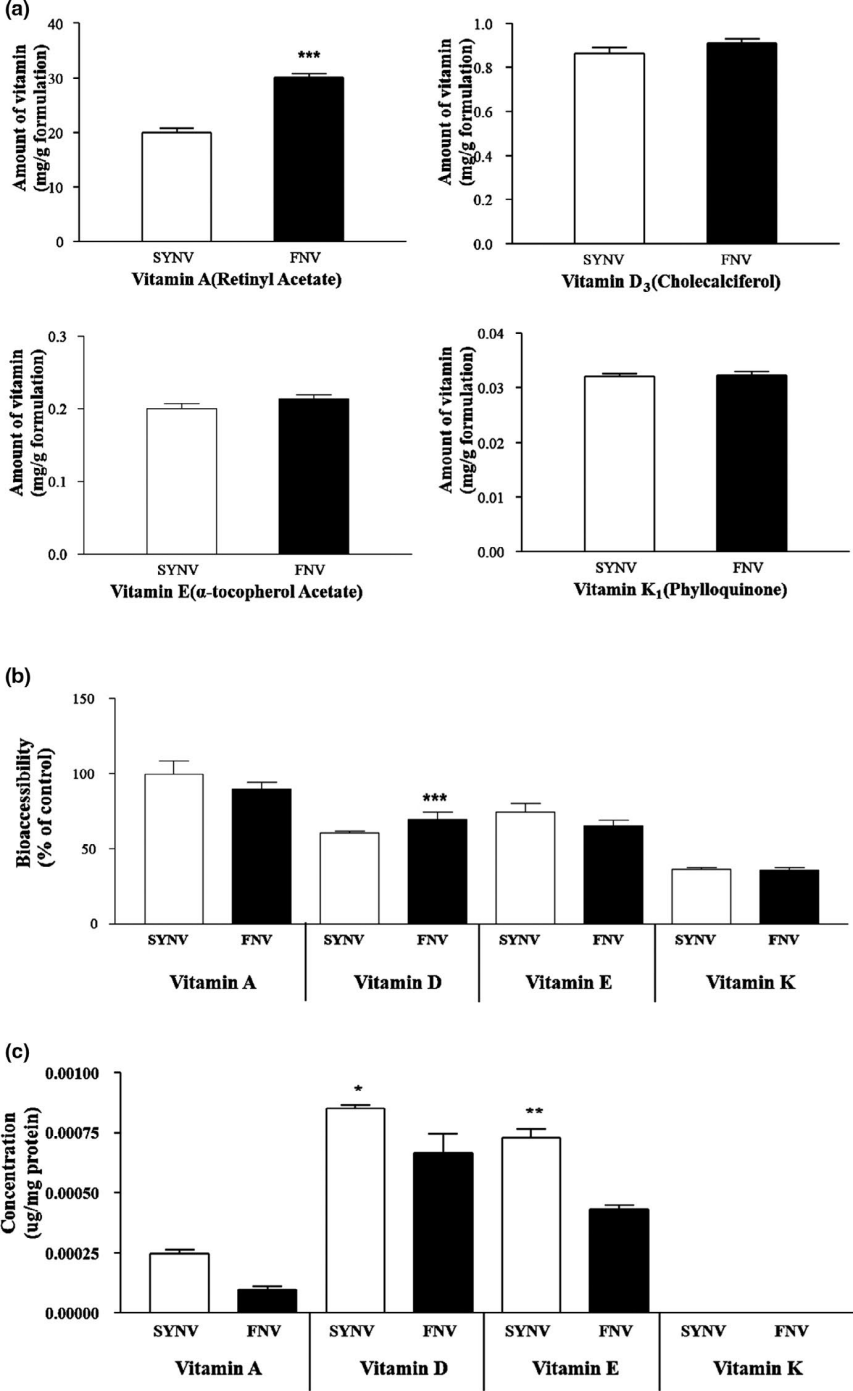
Determination of vitamins A, D, E, and K in FNV (a), bio‐accessibility of FNV (b) and the intestinal cellular uptake of FNV (c). The data include the means ± 95% CI of the triplicated samples. An asterisk indicates a significant difference from the positive control (SYNV). (**p < .01; *p < .05)

The initial amount of vitamins A, D, E, and K from each FNV was calculated according to each calibration curve of retinyl acetate, cholecalciferol, alpha‐tocopherol acetate, and phylloquinone, respectively (Figure 4a). The FNV A, D, E, and K contained 30.129 mg/g, 0.910 mg/g, 0.214 mg/g, and 0.032 mg/g, respectively, which was approximately 1.5, 1.1, 1.1, and 1 times higher than that of the SYNV A, D, E, and K, respectively. To estimate their bioavailability, the bio‐accessibility and the intestinal cellular uptake were determined using the in vitro digestion model system coupled with a Caco‐2 cell. The bio‐accessibility was calculated by the relative percent of the content of the vitamin detected in the aqueous fraction followed by the digestion compared to the initial vitamin amount before digestion. The bio‐accessibility of the FNV A, D, E, and K was 89%, 73%, 65%, and 36%, respectively, which was comparable to the bio‐accessibility of the SYNV (Figure 4b). Excluding the vitamins D and K, the bio‐accessibility of the FNV A and the E (89% and 65%) were significantly lower than the SYNV A (99%) and the E (74%) (*p* < .05). The bio‐accessibility of the FNV D (73%) was significantly higher than the SYNV D (57%) (*p* > .05). However, there was no significant difference between the FNV K (36%) and the SYNV K (37%) (*p* > .05).

The Caco‐2 cellular uptake of the FNV was normalized by the cellular protein (µg/g protein), and it occurred in the following order: 0.0001 µg/g, 0.0007 µg/g, and 0.0004 µg/g for the FNV A, D, and E, which is illustrated in Figure 4c. In the case of the SYNV, 0.0002 µg/g of A, 0.0009 µg/g of D, and 0.0007 µg/g of E were up‐taken by the Caoc‐2 cell. Vitamin K was not detected in the Caco‐2 cell, which indicates that it was below the limit of detection (<0.05 mg/L). The absorption of the fat‐soluble vitamins from the gastrointestinal tract consists of several consecutive stages, which include the physicochemical and the enzymatic reactions (Borel, 2003). In particular, the bile acid played an essential role to facilitate the absorption of the fat‐soluble vitamins (Saeed, Dullaart, Schreuder, Blokzijl, & Faber, 2017; Setchell et al., 2013). A previous study reported that the higher bio‐accessibility of the fat‐soluble vitamin incorporation into the micelles provided greater absorption efficiency (Reboul, 2015). The micelles were generated by the lipolysis of the dietary fat where there are a mixture of phospholipids, cholesterol, lipid digestion products, and bile salts (Reboul, 2015). The FVN tablet, which was formulated by vitamins extracted from the agricultural products with synthetic vitamin in a 6 to 4 ratio (w:w), did not affect the digestive stability compared to the SYNV. This implies that the matrix from the agricultural products could be protected from the enzymatic reactions and formed micelle during the digestion. In contrast to the bio‐accessibility, the intestinal absorption of the FNV was lower than that of the SYNV, which could be affected by the dietary lipids and the intestinal transporters regulated by the components/matrix in the agricultural products that consisted of the FNV. Generally, the fat load was necessary to promote the chylomicron formation, because other dietary lipids enter the body with initial triglyceride‐rich chylomicron constituents (D’Ambrosio, Clugston, & Blaner, 2011; Kiela & Ghishan, 2016). In addition, the intestinal fat‐soluble vitamin absorption was determined by the control of several proteins that related to the intestinal transport (Harrison & Kopec, 2018). For example, the efficiency of vitamin A and the carotenoid intestinal absorption was controlled by the scavenger receptor class B type 1 (SR‐B1). It is assumed that vitamin A and the carotenoid could be comparatively absorbed by the different interactions with the active transporter proteins when they are absorbed at the same time by the intestinal membrane. In the same way, the fat‐soluble phytochemicals presented in the FNV could interrupt the binding of the fat‐soluble vitamins to a protein involved in the active transport process. Further studies to elucidate this mechanism should be conducted. In the case of vitamin K, which cannot be refluxed through the Caco‐2 cells, it is still unknown whether vitamin K is mediated by the other active transporters.

### 3.4 Prediction of shelf life for FNV during accelerated storage

The changes in the amount of the FNV A, D, E, and K at three temperatures, which included 25℃, 30℃, and 35℃, during storage are shown in Table 1. Overall, the vitamin content showed a decreasing tendency during storage in the FNV A, D, E, and K samples after accelerated storage for 6 months. In detail, the FNV A initially contained 148.83 ± 0.47 mg/g, and it significantly dropped to 22.85 ± 0.04 mg/g at 25℃, 27.27 ± 0.06 mg/g at 30℃, and 36.13 ± 0.06 mg/g at 35℃ after 6 months. The content of the FNV D was 1.66 ± 0.01 on day 0, 1.51 ± 0.01 for 6 months at 25℃6 months 1.52 ± 0.02 for 6 months at 30℃, and 1.54 ± 0.02 for 6 months at 35℃. In the case of the FNV E, it was 476.62 ± 0.56 on day 0, 420.77 ± 3.32 at 25℃, 427.89 ± 1.21 at 30℃, and 440.34 ± 0.69 at 35℃ during the 6 months. The amount of the FNV K was maintained at 100%, 77%, and 89% at 25℃, 30℃, and 35℃, respectively. Significant degradation was found in vitamin A that was stored at both 25℃ and 30℃, which had 15% and 18% remaining compared to 24% remaining at 35℃, which implies that the storage temperature for vitamin A was critical point rather than the oxygen and the humidity. The results were in agreement with the previous findings that reported that the storage length was by far the most decisive factor, and a negative correlation between vitamin A retention and storage time existed (Hemery et al., 2018; Wang, 2019). The stability of the FNV D and the E ranged from 91% to 93% and 88% to 92%, respectively, during the 6 months of storage. This finding was similar to the previous results, which vitamin D and vitamin E were not easily degraded by processing or during storage (Loewen et al., 2018; Raikos, 2017).

**Table 1:** Determination of the initial and the final contents of FNV A, D, E, and K at three temperatures

Sample	Temperature (°C)	Month					
		0	1.2	2.4	3.6	4.9	6
NFV A (mg/g)	25℃	148.83 ± 0.47	115.58 ± 1.13	48.09 ± 2.30	44.19 ± 2.11	34.8 ± 0.18	22.85 ± 0.04
	30℃		147.84 ± 0.97	50.62 ± 0.17	45.22 ± 6.72	37.91 ± 0.02	27.27 ± 0.05
	35℃		116.76 ± 2.46	68.19 ± 0.75	32.28 ± 0.32	26.28 ± 0.15	36.13 ± 0.05
NFV D (mg/g)	25℃	1.66 ± 0.00	1.58 ± 0.03	1.55 ± 0.01	1.64 ± 0.01	1.58 ± 0.05	1.51 ± 0.01
	30℃		1.55 ± 0.00	1.52 ± 0.00	1.58 ± 0.02	1.44 ± 0.01	1.52 ± 0.01
	35℃		1.56 ± 0.00	1.54 ± 0.00	1.51 ± 0.00	1.49 ± 0.00	1.54 ± 0.02
NFV E (mg/g)	25℃	476.62 ± 0.46	451.48 ± 0.60	441.55 ± 2.85	426.86 ± 1.58	421.22 ± 0.99	420.77 ± 2.71
	30℃		465.24 ± 0.74	444.42 ± 0.52	426.99 ± 2.74	423.81 ± 1.84	427.89 ± 0.99
	35℃		474.13 ± 1.18	446.20 ± 0.48	440.64 ± 0.83	436.59 ± 1.42	440.34 ± 0.56
NFV K (mg/g)	25℃	0.16 ± 0.00	0.15 ± 0.00	0.15 ± 0.00	0.15 ± 0.00	0.15 ± 0.00	0.16 ± 0.00
	30℃		0.14 ± 0.00	0.14 ± 0.00	0.15 ± 0.00	0.15 ± 0.00	0.12 ± 0.00
	35℃		0.15 ± 0.00	0.14 ± 0.00	0.15 ± 0.00	0.15 ± 0.00	0.14 ± 0.00

**Figure 5:**
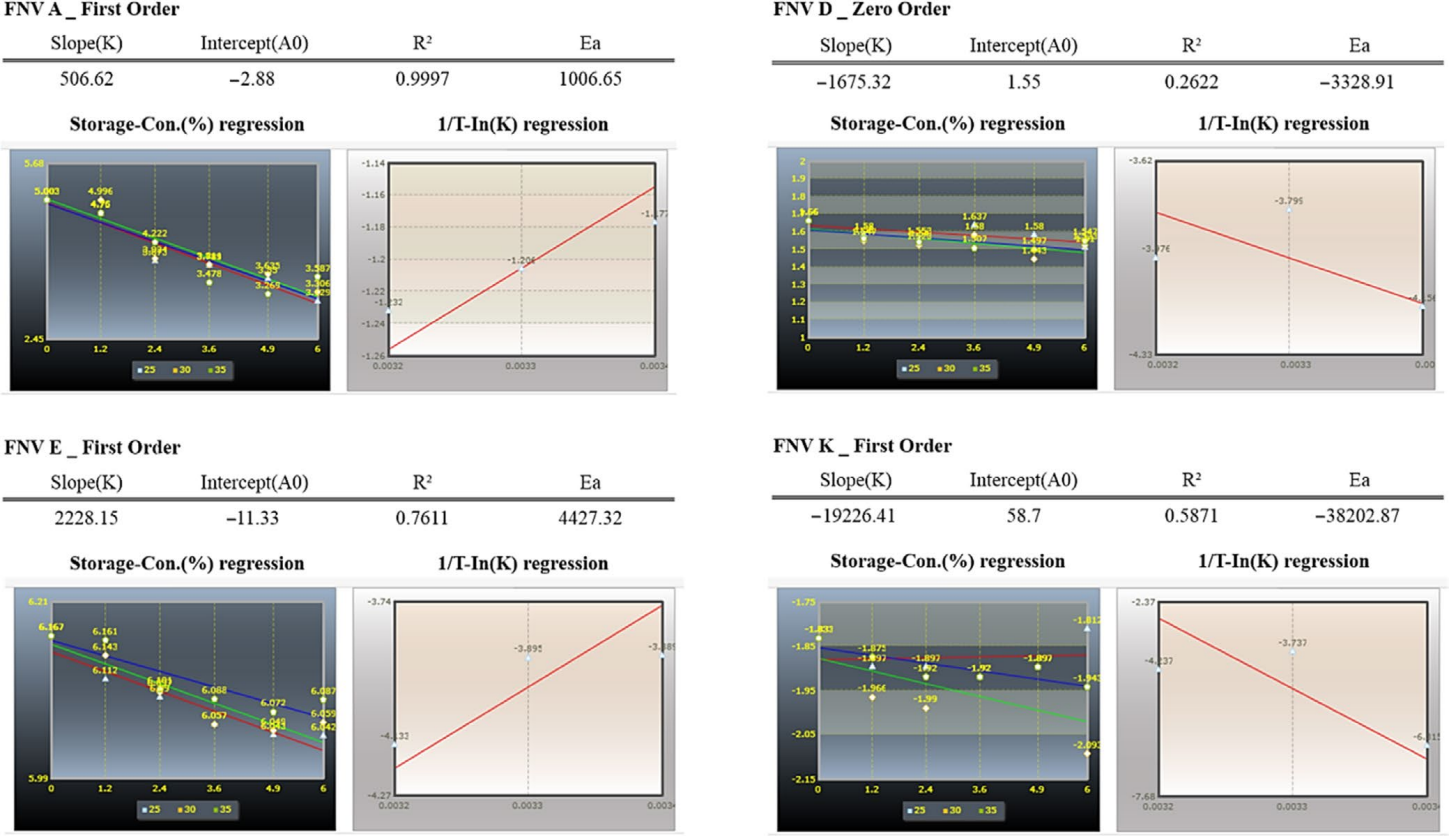
Relation between 1/T and ln k using the Arrhenius equation (activation energy)

To describe the temperature dependence of the reaction rate constant, the k values were used (Table 2), and they were plotted as linearity to the corresponding 1/T temperature. The obtained activation energy (Ea) and the k0 are shown in Figure 5. By combining the zero or the first‐order motion model, a storage time calculation model with the temperature (T) and the quality factor variables were also obtained as shown in Table 3. Considering the statistical analysis, which is based on the R_2_, it was found that the contents of the FNV A, E, and K followed the first‐order reaction, while the content of the FNV D fit the zero‐order reaction as than the first‐order reaction. Thus, each linear regression for the FNV A, E, and K was calculated by the first‐order equations, and the linear regression of the FNV D was calculated by the zero‐order equations that corresponded to the values of the k (reaction velocity). Finally, the final shelf life of the FNV A, D, E, and K was estimated for the temperature of 30℃, which considered the multiple of the shelf life by the safety factor of 0.8. Based on the measurement by the Arrhenius equation and the safety factor, it was estimated that the FNV A, D, E, and K have legal shelf lives of 0.6, 14.0, 11.4, and 7.4 months in Korea, respectively.

**Table 2:** Constant of kinetics rate of FNV A, D, E, and K in storage temperatures of 25, 30, and 35℃

Sample	Temperature (°C)	Order	Slope (K)	Intercept (A0)	*R* ^2^
NFV A (mg/g)	25℃	Zero Order	−20.694	131.4831	0.8491
	30℃		−22.2781	143.4885	0.7991
	35℃		−20.6089	133.5964	0.8442
	25℃	First Order	−0.3082	4.9451	0.9379
	30℃		−0.2994	5.0159	0.8892
	35℃		−0.2916	4.9329	0.8384
NFV D (mg/g)	25℃	Zero Order	−0.0157	1.6339	0.4198
	30℃		−0.0224	1.6136	0.498
	35℃		−0.0119	1.6082	0.5284
	25℃	First Order	−0.0099	0.491	0.4218
	30℃		−0.0144	0.4782	0.493
	35℃		−0.0119	0.4746	0.5282
NFV E (mg/g)	25℃	Zero Order	−9.1008	467.2053	0.8935
	30℃		−9.123	471.6832	0.8609
	35℃		−7.3126	473.9802	0.7889
	25℃	First Order	−0.0205	6.147	0.9022
	30℃		−0.0203	6.1565	0.8623
	35℃		−0.016	6.1612	0.7903
NFV K (mg/g)	25℃	Zero Order	0.0003	0.1524	0.0103
	30℃		−0.0033	0.1528	0.3614
	35℃		−0.0022	0.1566	0.6899
	25℃	First Order	0.0018	−1.8814	0.0091
	30℃		−0.0238	−1.8779	0.3645
	35℃		−0.0145	−1.8542	0.6914
NFV A (mg/g)	25℃	Zero Order	−20.694	131.4831	0.8491
	30℃		−22.2781	143.4885	0.7991
	35℃		−20.6089	133.5964	0.8442
	25℃	First Order	−0.3082	4.9451	0.9379
	30℃		−0.2994	5.0159	0.8892
	35℃		−0.2916	4.9329	0.8384
NFV D (mg/g)	25℃	Zero Order	−0.0157	1.6339	0.4198
	30℃		−0.0224	1.6136	0.498
	35℃		−0.0119	1.6082	0.5284
	25℃	First Order	−0.0099	0.491	0.4218
	30℃		−0.0144	0.4782	0.493
	35℃		−0.0119	0.4746	0.5282
NFV E (mg/g)	25℃	Zero Order	−9.1008	467.2053	0.8935
	30℃		−9.123	471.6832	0.8609
	35℃		−7.3126	473.9802	0.7889
	25℃	First Order	−0.0205	6.147	0.9022
	30℃		−0.0203	6.1565	0.8623
	35℃		−0.016	6.1612	0.7903
NFV K (mg/g)	25℃	Zero Order	0.0003	0.1524	0.0103
	30℃		−0.0033	0.1528	0.3614
	35℃		−0.0022	0.1566	0.6899
	25℃	First Order	0.0018	−1.8814	0.0091
	30℃		−0.0238	−1.8779	0.3645
	35℃		−0.0145	−1.8542	0.6914

**Table 3:** Prediction of the shelf life of the samples FNV A, D, E, and K

Sample	Order	Initial content specifications	Reaction rate constant	Shelf life (months)	Final shelf life^a^ (months)
NFV A	First Order	0.2279	3.5	0.78	0.62
NFV D	Zero Order	0.36	0.25	17.55	14.04
NFV E	First Order	0.2371	0.2	14.21	11.37
NFV K	First Order	0.2231	0.29	9.22	7.38

^a^ Final shelf life = Estimated shelf life (month)×Safety factor (0.8).

A reliable shelf‐life assessment for functional foods was suggested to be critical to verify how much vitamins will last in order to provide the proper bioactivity, which depends on the preferences of the customers (Calligaris, Manzocco, Anese, & Nicoli, 2016). Based on these results, some complementary ways could be recommended in order to optimize the retention of the vitamins in the FNV A, D, E, and K by modulating the exposure time to air as short as possible in order to delay or reduce the oxidation and the degradation reactions. Hence, the current study measured the radical scavenging capacity of the FNV in order to examine its oxidation during storage.

### 3.5 Radical scavenging capacity of FNV

**Figure 6:**
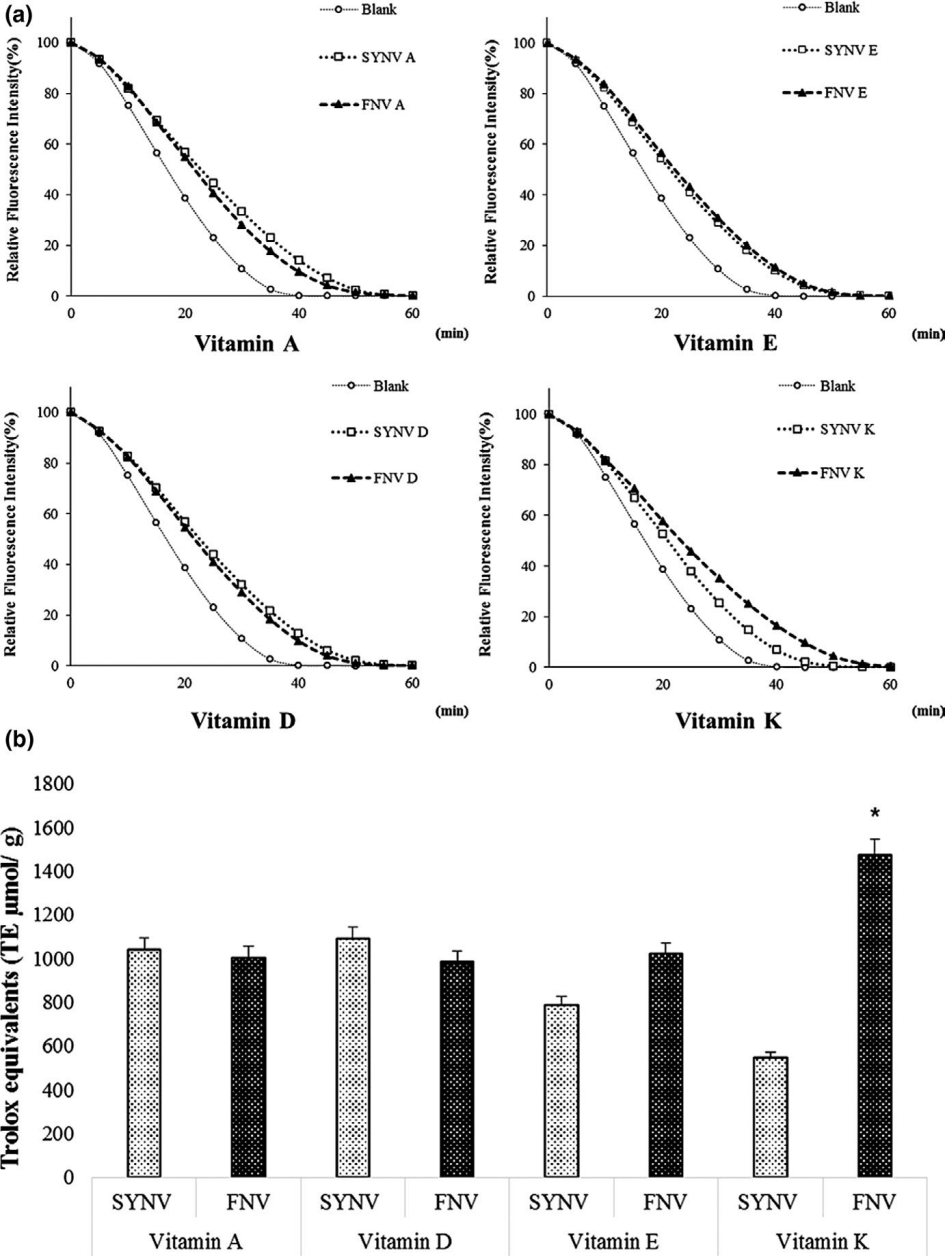
Effect of FNV A, D, E, and K on the peroxy radical (ROO•) scavenging capacity. (a), relative fluorescence intensity (%) according to time (b) and the Trolox equivalent (TE, μmol) based on the area under the curve

Figure 6a exhibits the changes in the value of fluorescence over time and the extent of the oxygen radical absorbance capacity (ORAC) of the FNV. It was found that the AUC extent to the curves of the relative fluorescence intensity of the FNV A, D, E, and K was not significantly different between the FNV and the SYV tablets (*p* < .05). The ORAC value for the FNV A (1,004.92 µmol TE/g) and the D (983.42 µmol TE/g) was slightly lower than that of the SYV, but it was not significantly different from the SYV A and the D (*p* > .05) (Figure 5b). However, the FNV E and the K showed 1,019.71 µmol TE/g and 1,472.85 µmol TE/g, which indicates that it was higher than the SYN E (785.91 µmol TE/g) and the K (546.26 µmol TE/g). Dietary antioxidants, which are predominantly found in fruits and vegetables, were used to prevent the process of oxidation and the chemical reaction promoted by the reactive oxygen species, such as oxygen peroxide (Park et al., 2017; Shinagawa, Santana, Araujo, Purgatto, & Mancini‐Filho, 2018). A previous study found that fresh carrots and spinach had 1.93 µmol TE/g and 28.14 µmol TE/g of ORAC (Shiwakoti, Zheljazkov, & Schlegel, 2018). The results from the current study found that the FNV A and the K had 520 and 52 times stronger antioxidant capacity, respectively. In the case of the ORAC for the FNV D (983.42 µmol TE/g), it exceeded 9 times the amount found in mushrooms (113.9 µmol TE/g), which are known as an exceptional source of this nutrient (Smolskaitė, Venskutonis, & Talou, 2015). The effect of the ORAC for the FNV E (1,019.7 µmol TE/g) was about 317 times higher than a previous finding on fresh tomatoes (3.22 µmol TE/g) (Köstekli et al., 2016). Taken in conjunction, the FNV mainly used by agricultural products could be the optimal natural antioxidants needed to contribute to human health by reducing the free radicals.

## 4 CONCLUSIONS

The fat‐soluble vitamins A, D, E, and K were extracted from carrots, mushrooms, tomatoes, and spinach, in order to formulate natural vitamins (FNV), which was followed by formulating with synthetic vitamins with 6 to 4 ratios. The FNV was well dispersed in water without a crystal form, which indicates that it was within the dispersion specifications for a functional table for foods. The bio‐accessibility of the FNV D was higher than the bio‐accessibility of the SYNV D, but the FNV A, E, and K were not significantly different than that of the SYNV A, E, and K, which was followed by the in vitro digestion. However, the intestinal absorption by the Caco‐2 cell of the FNV, which included the FNV D, was lower than the SYNV. The FNV A degradation was high and occurred rapidly, but the FNV D, E, and K contents did not significantly change during the 6 months storage time under the same storage conditions. The changes of the radical scavenging capacity in the FNV A, D, E, and K were comparable to those of the SYNV. The results from the current study suggest that the FNV A, D, E, and K could be used as complementary ingredients for the natural vitamin supplements.

## CONFLICT OF INTEREST

The authors declare that they have no conflicts of interest.
